# Supplementary figures and images for: Current Overview of Spinocerebellar Ataxia Type 7 in Mexican Population: Challenges in Specialized Care for a Rare Disease
Source: Int J Mol Sci. 2024 Oct 6;25(19):10750. doi: 10.3390/ijms251910750 (PMC11476844; doi:10.3390/ijms251910750)

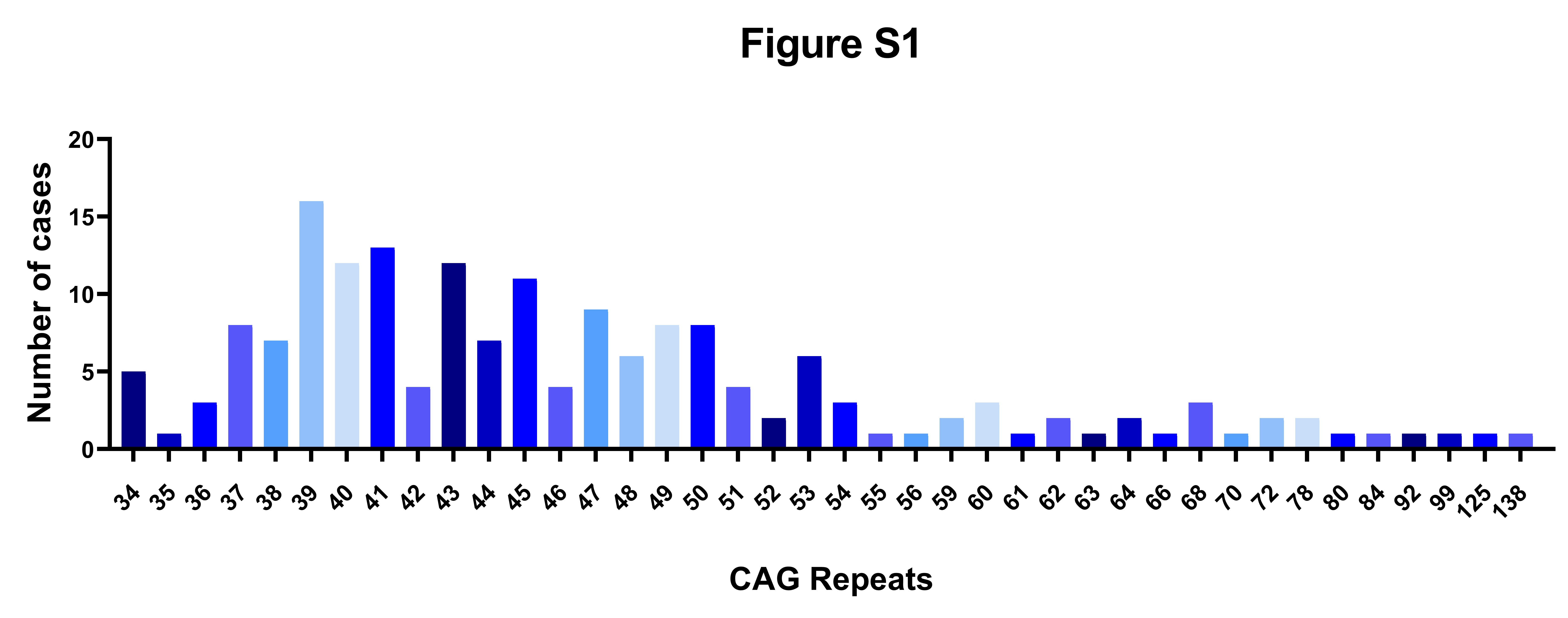

Supplement: Supplementary file 1 [file ijms-25-10750-s001.zip › ijms-3211659-supplementary.jpg]
